# Glucocorticoid-Free Induction Therapy With Hydroxychloroquine and Anifrolumab in Systemic Lupus Erythematosus: A Case Report

**DOI:** 10.7759/cureus.100230

**Published:** 2025-12-27

**Authors:** Mitsuki Tezuka, Shimoyama Shuhei, Masanari Sugawara, Keisuke Kikuchi, Yayoi Ogawa, Mitsuru Yanai, Yuka Shimizu

**Affiliations:** 1 Department of Rheumatology, Obihiro-Kosei General Hospital, Obihiro, JPN; 2 Department of Pathology, Obihiro-Kosei General Hospital, Obihiro, JPN; 3 Department of Diagnostic Pathology, Sapporo Higashi Tokushukai Hospital, Sapporo, JPN

**Keywords:** anifrolumab, glucocorticoid-free remission, hydroxychloroquine (hcq), interferon (ifn), systemic lupus erythematosus

## Abstract

Systemic lupus erythematosus (SLE) is a chronic autoimmune disorder typically managed with glucocorticoids (GCs), but there are significant risks associated with their long-term use. With the new immunosuppressive agents and biologics, discontinuation of GC usage while effectively controlling disease flares has become feasible. But GC-free induction therapy in SLE remains challenging. Here, we show a 60-year-old woman diagnosed with SLE, who successfully managed her disease without GCs, using hydroxychloroquine (HCQ) and anifrolumab (ANI) as primary treatment options. The patient initially presented with symptoms including facial erythema, fever, leukocytopenia, thrombocytopenia, anaemia, proteinuria, and lymphadenitis, raising suspicion of an SLE flare. Laboratory tests and imaging confirmed the diagnosis, and a lymph node biopsy revealed necrotising lymphadenitis. A renal biopsy indicated class II lupus nephritis. Despite initial treatment with HCQ, the patient's condition did not improve. We sequentially added ANI, resulting in rapid resolution of fever, improvement in blood counts, and significant reduction in rash severity. Importantly, she did not need GCs to treat SLE. ANI is a monoclonal antibody targeting the type I interferon receptor subunit 1 and can be a key drug for SLE. The findings suggest that GC-free induction therapy can be an approach for controlling disease activity in SLE.

## Introduction

Systemic lupus erythematosus (SLE) is a chronic autoimmune disorder characterized by multi-organ involvement and variable disease activity. For decades, glucocorticoids (GCs) have been the mainstay of therapy to control disease flares [1]. However, prolonged GC exposure is associated with serious adverse effects such as osteoporosis, cardiovascular disease, metabolic complications, and increased infection risk [2,3]. Reflecting these concerns, the 2023 European Alliance of Associations for Rheumatology (EULAR) recommendations emphasize minimizing GC use whenever possible, advising that GCs be administered “if needed” and tapered early with a goal of discontinuation once disease control is achieved [4].

In recent years, new immunosuppressive agents and biologics have expanded treatment options. Hydroxychloroquine (HCQ) is recommended for almost all patients if no safety concerns are present, as it reduces flare frequency and improves long-term survival [5,6]. Furthermore, anifrolumab (ANI), a monoclonal antibody against the type I interferon receptor, has shown significant efficacy in clinical trials, particularly for skin, musculoskeletal, and hematologic manifestations [7]. These developments have led to increasing interest in GC-sparing or even GC-free treatment strategies, though real-world reports of GC-free induction therapy remain scarce, and the feasibility of such approaches is not fully established.

Given this evolving therapeutic landscape, carefully selected patients, such as those without organ-threatening disease, may be candidates for sequential introduction of non-GC agents while reserving GCs for inadequate response. This approach is consistent with EULAR 2023 guidance, which does not prohibit initiating therapy without GCs, provided that steroids are added promptly if disease activity persists or worsens.

Here, we report a patient with SLE presenting with fever, facial rash, cytopenias, necrotizing lymphadenitis, and class II lupus nephritis who achieved remission using HCQ and ANI without systemic GCs. This case provides real-world evidence supporting the possibility of GC-free induction therapy in selected patients aligned with modern GC-minimization principles.

## Case presentation

A 60-year-old woman with no significant medical history developed loss of appetite in October 2024, followed two weeks later by persistent fever up to 38.5°C, sore throat, and a progressive malar rash. She had not been taking systemic medications prior to presentation, aside from occasional use of a low-potency topical corticosteroid (hydrocortisone 1%) for symptomatic relief of the facial rash. She denied arthralgia, serositis, neurologic symptoms, oral ulcers, or other organ-threatening manifestations.

**Figure 1:**
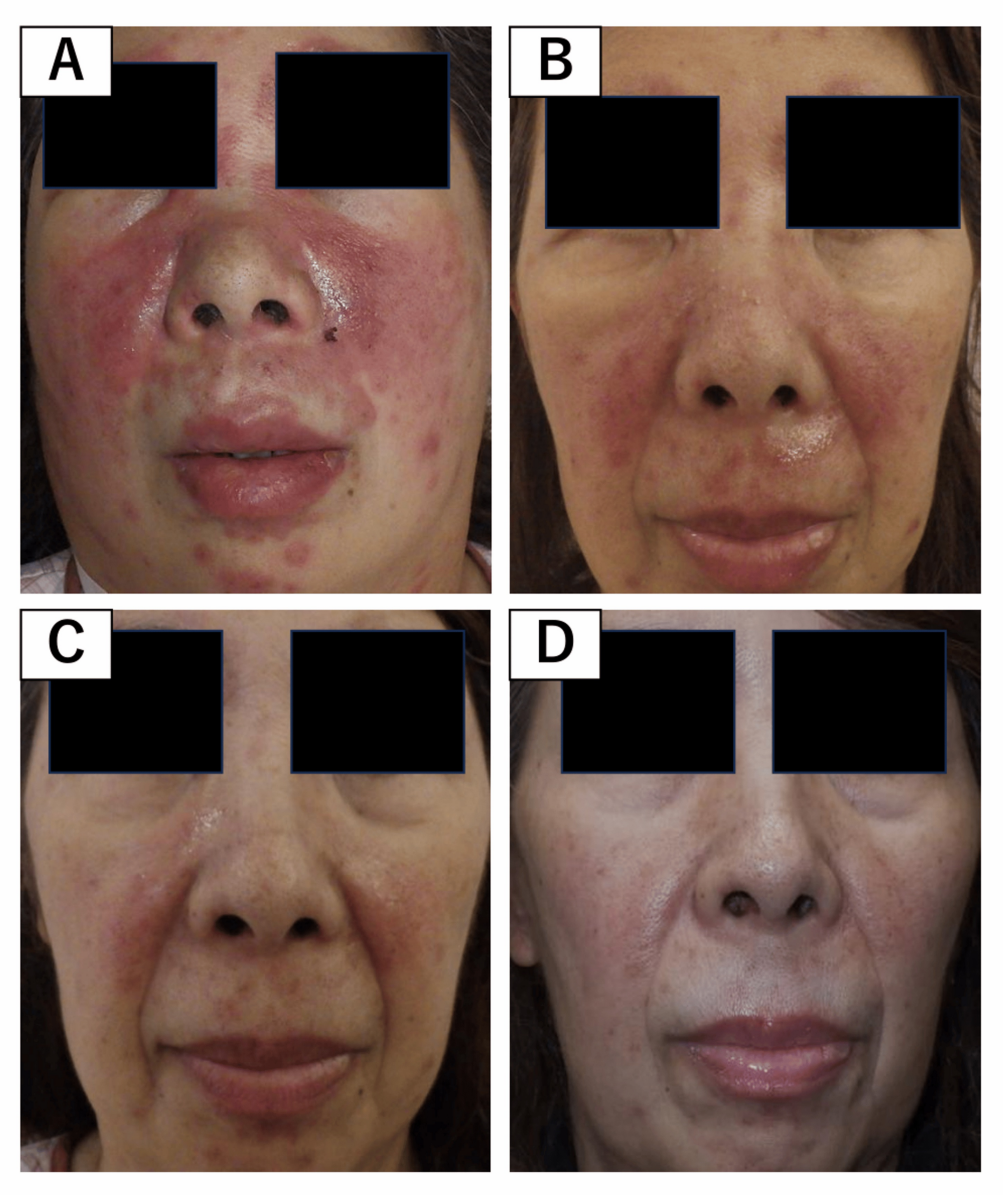
Clinical images of facial rash. Serial photographs showing gradual resolution of the malar rash after treatment with anifrolumab: (A) Day 2, (B) day 9, (C) day 15, and (D) day 29. Written informed consent was obtained from the patient for publication.

On physical examination, she exhibited a well-demarcated malar rash extending across the cheeks and nasal bridge and a tender right cervical lymph node. No synovitis, peripheral edema, or mucosal ulcers were observed. Serial clinical images documenting rash progression and improvement are shown in Figure 1.

Initial laboratory tests revealed cytopenias with worsening trends during early observation. The most severe abnormalities included leukopenia (white blood cell count 1.1 × 10^9^/L), anemia (hemoglobin 9.9 g/dL), and thrombocytopenia (platelet count 7.6 × 10^9^/L) (Table 1). Complement levels were markedly reduced (complement component 3 (C3) 52 mg/dL, complement component 4 (C4) 6 mg/dL, total hemolytic complement (CH50) 21.5 U/mL), and anti-double-stranded DNA antibody (anti-dsDNA) levels were elevated. Antinuclear antibody (ANA) testing was positive with a high titer by indirect immunofluorescence assay (IFA). Urinalysis initially showed mild proteinuria (urine protein-to-creatinine ratio (UPCR) 0.4 g/gCr) without hematuria.

**Table 1:** Pertinent laboratory results. The ANA value represents the titer determined by indirect immunofluorescence. The patient’s proteinuria increased from 0.4 g/gCr at the first visit to 0.8 g/gCr at the time of biopsy. H indicates a value above the reference range (high), and L indicates a value below the reference range (low). WBC: white blood cells; RBC: red blood cells; MCV: mean corpuscular volume; MCH: mean corpuscular hemoglobin; MCHC: mean corpuscular hemoglobin concentration; ANA: antinuclear antibody; IFA: immunofluorescence assay. Sm: Smith; ds: double-stranded; anti-SS-A antibody: anti-Sjögren’s syndrome-related antigen A (Ro) antibody; C3: complement component 3; C4: complement component 4; CH50: total hemolytic complement

Test	Results	Normal range	Units
WBC	1.1 (L)	3.3-8.6	Thousand per microliter
RBC	3.75 (L)	3.86-4.92	Million per microliter
Hemoglobin	9.9 (L)	11.6-14.8	g/dL
Hematocrit	30.3 (L)	35.1-44.4	%
MCV	80.8 (L)	83.6-98.2	fL
MCH	26.4 (L)	27.5-33.2	pg
MCHC	32.7	31.7-35.3	g/dL
Platelet	7.6 (L)	158-348	Thousand per microliter
C3	52 (L)	73-138	mg/dL
C4	6 (L)	11-31	mg/dL
CH50	21.5 (L)	31.6-57.6	U/mL
ANA screen IFA	640 (H)	0-39	-
Homogenous	640 (H)	0-39	-
Speckled	640 (H)	0-39	-
Anti-Sm antibody	1.9	0-9.9	-
Anti-dsDNA antibody	51 (H)	0-12	IU/mL
Anti-SS-A antibody	118 (H)	0-9.9	U/mL
lupus anticoagulant	1.1	0-1.2	-
Anti-cardiolipin antibody IgM	13	0-20.8	U/mL
Anti-beta-2-glycoprotein I antibody IgM	2	0-17.5	U/mL
Urinary protein	1+	-	-
Urinary blood	Negative	-	-
Urinary protein	0.8	-	g/gCr

**Figure 2:**
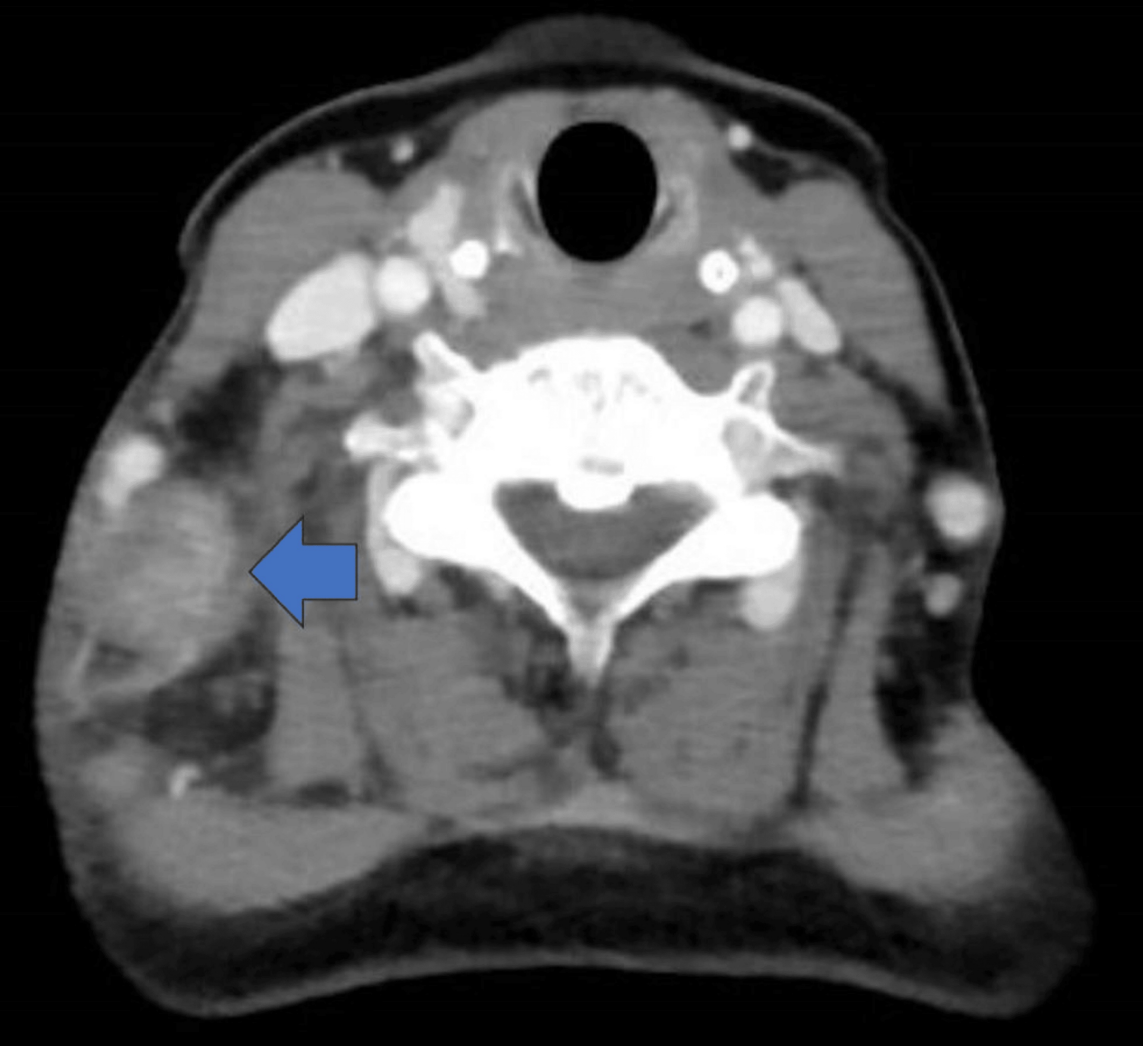
CT image of the neck. Contrast-enhanced cervical CT demonstrating enlargement of the right cervical lymph node (arrow). Axial view; slice thickness 5 mm.

**Figure 3:**
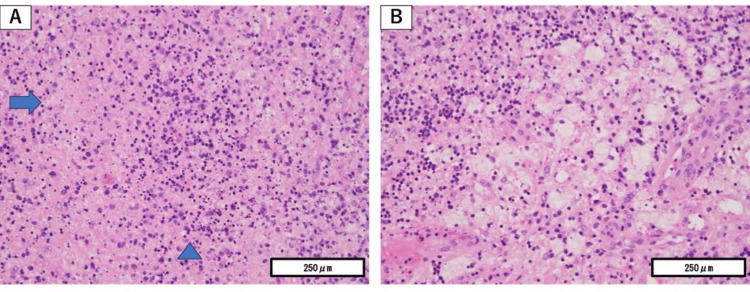
Lymph node biopsy findings. Hematoxylin and eosin (H&E)-stained light microscopy images. (A) Low-power view showing scattered necrotic areas (arrows) containing abundant nuclear debris (arrowheads). (B) High-power view demonstrating prominent macrophage infiltration surrounding the necrotic regions. Magnification: ×40. Findings are consistent with subacute necrotizing lymphadenitis.

Imaging demonstrated right-sided cervical lymphadenopathy (Figure 2). A cervical lymph-node biopsy revealed necrotizing lymphadenitis characterized by patchy necrosis with nuclear debris and macrophage infiltration, as shown in Figure 3.

**Figure 4:**
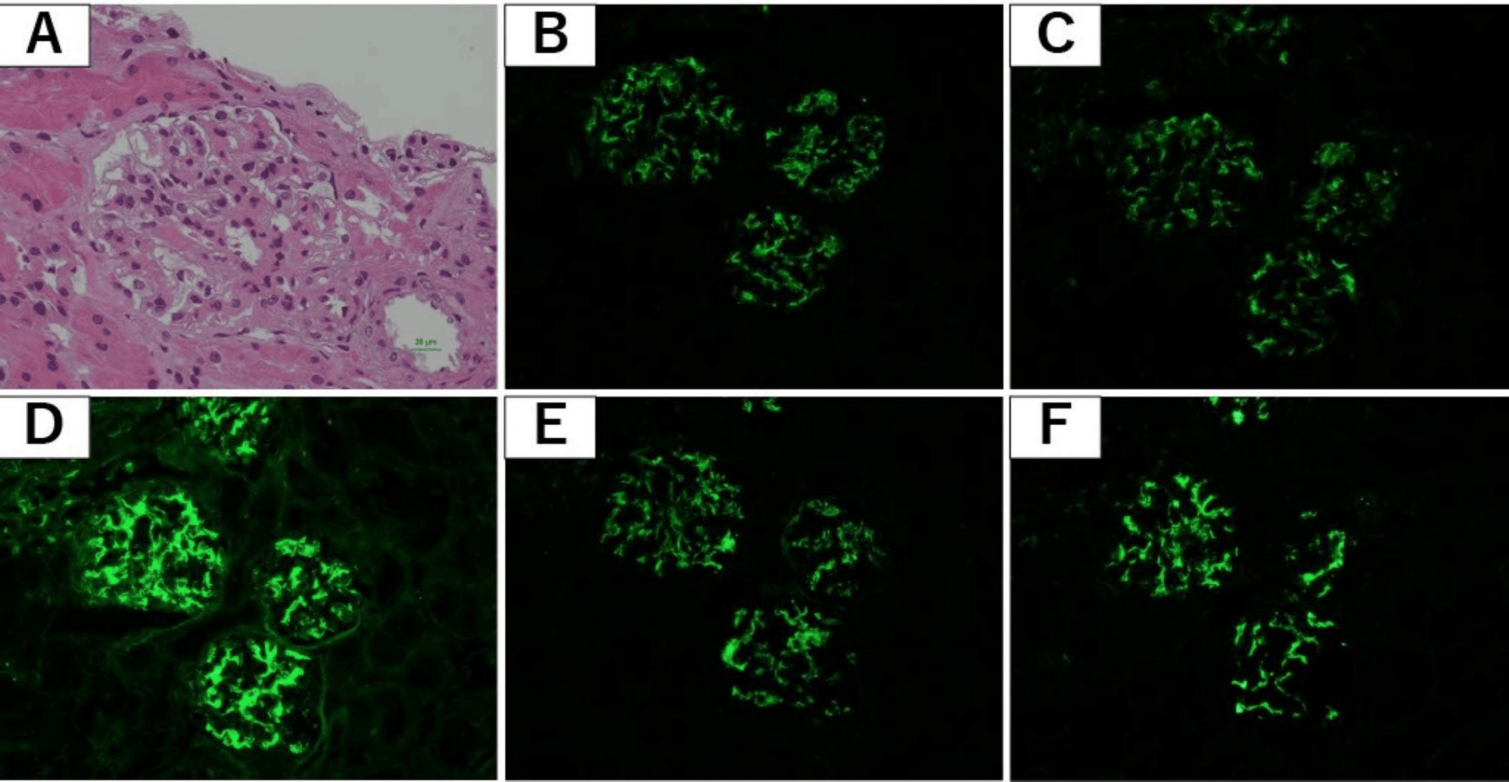
Renal biopsy findings (light microscopy and immunofluorescence). (A) Hematoxylin and eosin (H&E) staining (×400) demonstrating mild mesangial hypercellularity without crescentic or segmental lesions. (B-F) Immunofluorescence staining (×200) showing granular glomerular deposition of (B) IgA, (C) IgM, (D) IgG, (E) C3, and (F) C1q. Findings are consistent with class II lupus nephritis.

**Figure 5:**
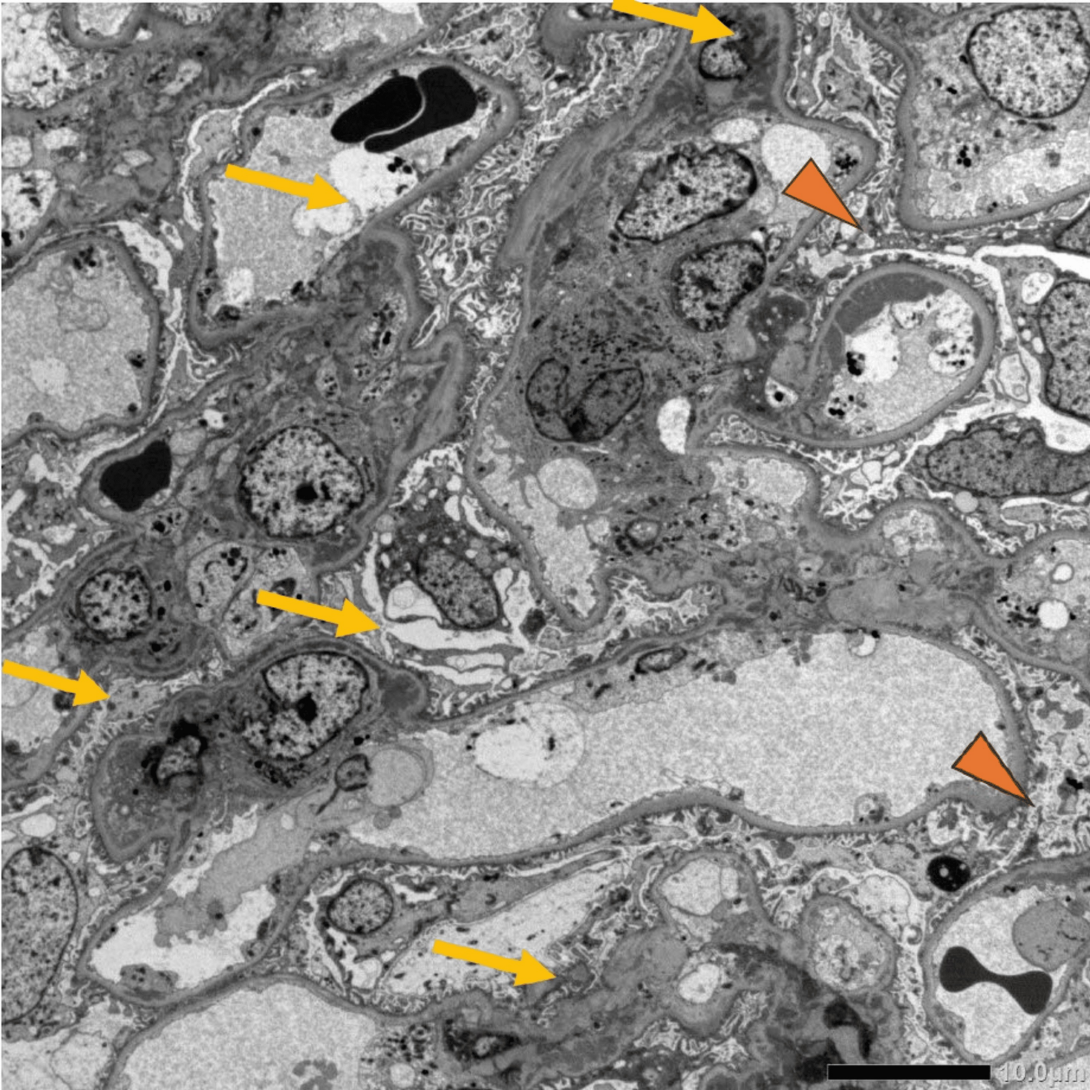
Renal biopsy findings on electron microscopy. Electron microscopy reveals mesangial and paramesangial electron-dense deposits (arrows) and additional subendothelial deposits (arrowheads). Magnification: ×8,000.

During the first week of observation, proteinuria increased to 0.8 g/gCr, surpassing the commonly used threshold for renal evaluation in mild SLE and showing a progressive trend. A renal biopsy was therefore performed. Light microscopy showed mild mesangial hypercellularity without crescentic or segmental lesions, and immunofluorescence demonstrated granular deposition of IgG, IgA, IgM, C3, and C1q, consistent with class II lupus nephritis (Figure 4). Electron microscopy confirmed mesangial and paramesangial electron-dense deposits with focal subendothelial involvement (Figure 5). The SLE Disease Activity Index 2000 (SLEDAI-2K) score at presentation was 10, indicating active but non-organ-threatening disease with mucocutaneous, constitutional, hematologic, and renal components.

Following diagnosis, HCQ 400 mg/day was initiated. However, fever and rash persisted, and cytopenias did not improve. Because the patient had no organ-threatening features and in accordance with EULAR 2023 guidance that GCs should be used “if needed,” the treating team adopted a sequential, safety-conscious therapeutic approach. Systemic GCs were initially deferred, with a plan for prompt initiation if HCQ proved insufficient.

**Figure 6:**
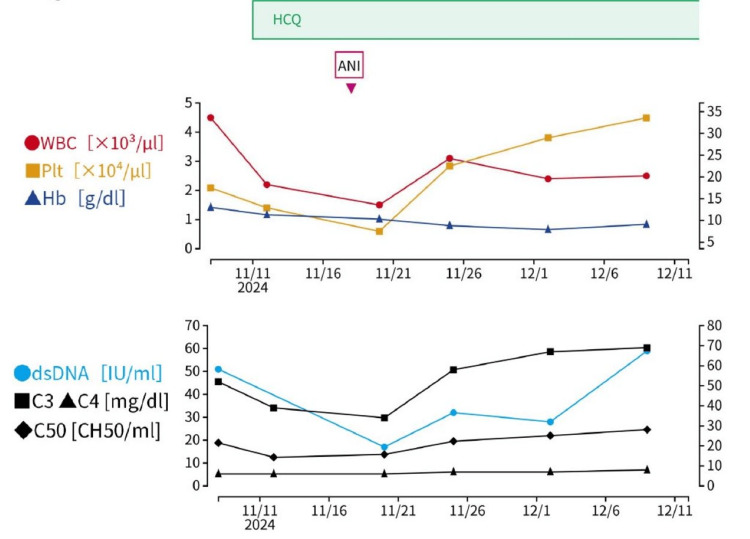
Sequential changes in hematologic and immunologic parameters following treatment. Line graphs illustrate temporal trends in (A) WBC count, Hb, and platelet count, and (B) immunologic markers including anti-dsDNA antibody, complement C3, complement C4, and CH50. Day 0 represents the day of anifrolumab administration. Hematologic abnormalities present at baseline (leukopenia, thrombocytopenia, and mild anemia) improved progressively after treatment. Similarly, anti-dsDNA antibody levels decreased, and complement levels (C3, C4, and CH50) showed gradual recovery, consistent with overall disease activity control. WBC: white blood cell count; Hb: hemoglobin; Plt: platelet count; dsDNA, double-stranded DNA; C3: complement component 3; C4: complement component 4; CH50: total hemolytic complement; HCQ: hydroxychloroquine; ANI: anifrolumab

ANI 300 mg IV was introduced (Day 0 of Figure 6). Fever resolved by Day 3, leukocyte and platelet counts improved by Day 4, and the malar rash progressively regressed over the following weeks (Figure 1). Complement levels (C3, C4, CH50), anti-dsDNA antibody titers, and proteinuria subsequently improved in parallel. These sequential hematologic and immunologic responses are summarized in Figure 6. Given the sustained multisystem improvement, systemic GCs were not required, and remission was achieved with HCQ and ANI alone.

## Discussion

The effective management of SLE requires early recognition and appropriate control of disease activity. Although GCs have historically been the cornerstone of induction therapy [1], prolonged exposure is strongly associated with irreversible organ damage, cardiovascular and metabolic complications, and increased infection risk [2,3]. Reflecting these concerns, the 2023 EULAR recommendations emphasize minimizing GC use and administering them only “if needed,” with a goal of reducing the dose to <5 mg/day or discontinuing entirely when possible [1,4]. In this context, immunosuppressive agents and biologics have become increasingly important components of GC-sparing strategies.

HCQ remains the foundational therapy in SLE because it reduces flare frequency and improves long-term outcomes [5,6]. In the present case, however, HCQ alone was insufficient to control the patient’s fever, facial rash, and hematologic abnormalities, prompting the addition of ANI. In the TULIP-2 trial, ANI demonstrated significant efficacy, particularly for type I interferon-mediated manifestations, including rash, constitutional symptoms, and cytopenias [7], which is consistent with the rapid improvement observed in this patient. Sequential laboratory measurements and the SLEDAI-2K score further supported the objective improvement following ANI initiation.

The patient’s renal involvement was mild, with biopsy-proven class II lupus nephritis and no evidence of organ-threatening disease. This clinical profile was central to the decision to initially defer systemic GCs. In addition to aligning with the GC-minimization principles of EULAR [1], a staggered therapeutic introduction is common clinical practice when multiple new agents are considered, as it allows clearer attribution of potential adverse effects. Importantly, GCs would have been introduced promptly had HCQ and ANI proved insufficient. Thus, the GC-free course in this patient reflects an individualized, safety-conscious approach rather than a generalizable treatment paradigm.

While the patient achieved steroid-free remission, this outcome should not be extrapolated to individuals with more severe lupus nephritis (class III-V) or organ-threatening SLE, for whom GCs remain an essential component of induction therapy [1]. Additionally, although necrotizing lymphadenitis may have contributed to disease activation, the temporal association between ANI administration and the rapid, multisystem improvement makes spontaneous remission or biopsy effect unlikely as sole explanations. Nevertheless, as a single case with mild disease, the findings should be interpreted cautiously.

This report highlights that, in selected patients with non-organ-threatening SLE, GC-free induction may be feasible when supported by close monitoring and timely access to biologic therapy. However, prospective studies and long-term follow-up are needed to determine which patient subsets may safely benefit from steroid-free approaches and to establish evidence-based guidance for the integration of biologics into GC-minimizing treatment strategies.

## Conclusions

In this case of non-organ-threatening SLE with class II lupus nephritis, disease activity was controlled without systemic GCs through the sequential use of HCQ and ANI. This approach was selected based on the patient’s mild clinical presentation and the need to evaluate treatment response in a stepwise and safety-conscious manner. The patient experienced rapid improvement in fever, cytopenias, and cutaneous manifestations shortly after initiating ANI, and remission was achieved without the introduction of systemic GCs.

This outcome should be interpreted cautiously. Steroid-free induction is not standard practice and is unlikely to be appropriate for patients with more severe lupus nephritis or organ-threatening disease. While GC avoidance may be feasible in carefully selected mild cases, further studies and additional clinical experience are needed to clarify the safety, reproducibility, and broader applicability of steroid-free induction strategies in SLE.
